# Bariatric surgery after childbirth: does a history of pregnancy affect weight loss after surgery? The Maternal Outcomes of Bariatric Surgery and Pregnancy Study (MOMBARIS)

**DOI:** 10.20452/wittm.2026.18009

**Published:** 2026-01-21

**Authors:** Piotr Małczak, Monika Malska, Anna Różańska -Walędziak, Nina Skalska -Dziobek, Kamila Siuda, Maciej Walędziak, Jacek Szeliga, Natalia Dowgiałło -Gornowicz, Bartosz Katkowski, Paula Franczak, Michał Janik, Michał Wysocki, Anna Kloczkowska, Piotr Major

**Affiliations:** Second Department of General Surgery, Jagiellonian University Medical College, Kraków, Poland; Department of Nursing, Institute of Health Sciences, University of the National Education Commission, Kraków, Poland; Faculty of Medicine, Collegium Medicum, Cardinal Stefan Wyszynski University in Warsaw, Warszawa, Poland; Department of General, Oncological, Metabolic, and Thoracic Surgery, Military Institute of Medicine -National Research Institute, Warszawa, Poland; Department of General, Gastroenterological, and Oncological Surgery, Collegium Medicum Nicolaus Copernicus University, Toruń, Poland; Department of General, Minimally Invasive, and Elderly Surgery University of Warmia and Mazury in Olsztyn Olsztyn Poland; Department of General and Vascular Surgery, Specialist Medical Center, Polanica -Zdrój, Poland; Department of General and Oncological Surgery, Ceynowa Hospital, Wejherowo, Poland; General Surgery Department Military Institute of Aviation Medicine Warszawa Poland; Department of General Surgery and Surgical Oncology, Ludwik Rydygier Memorial Hospital in Krakow, Kraków, Poland; Department of General, Endocrine, and Transplant Surgery, Faculty of Medicine Medical University of Gdansk Gdańsk Poland

**Keywords:** bariatric surgery, obesity, pregnancy, weight loss

## Abstract

**INTRODUCTION:**

Obesity is a major global health issue associated with comorbidities, such as type 2 diabetes mellitus (T2DM) and cardiovascular disease. Bariatric surgery is effective, but its outcomes vary. Obstetric history may influence results, as pregnancy induces lasting metabolic and hormonal changes, though current evidence remains unclear.

**AIM:**

This study aimed to evaluate whether preoperative pregnancy history affects weight loss outcomes after bariatric surgery.

**MATERIALS AND METHODS:**

A retrospective multicenter analysis was conducted within the Maternal Outcomes of Bariatric Surgery and Pregnancy Study project, including 1399 women from 11 Polish bariatric centers. The participants were divided into 2 groups: women with a history of pregnancy (n=1061) and nulliparous women (n=338). Primary outcomes included percentage of total weight loss (%TWL), percentage of excess weight loss (%EWL), and overall weight reduction.

**RESULTS:**

Women with prior childbirth were older (42 vs 32.5 y; *P* <⁠0.001) and more frequently had T2DM (22% vs 12%; *P* <⁠0.001) and hypertension (44.9% vs 23.4%; *P* <⁠0.001) than the nulliparous participants. Median postoperative body mass index (BMI) was similar in both groups (29 kg/m²), but weight loss differed considerably. Women with childbirth history achieved lower %TWL (28.57% vs 33.85%; *P* <⁠0.001) and %EWL (72.17% vs 78.44%; *P* =0.001), as compared with those who never gave birth. Multivariate regression identified age, preoperative BMI, hypertension, and dyslipidemia as independent factors affecting weight loss.

**CONCLUSIONS:**

Women with a history of childbirth achieve poorer weight loss outcomes after bariatric surgery; however, it is not an independent factor influencing bariatric results.

**FIGURE 1:**
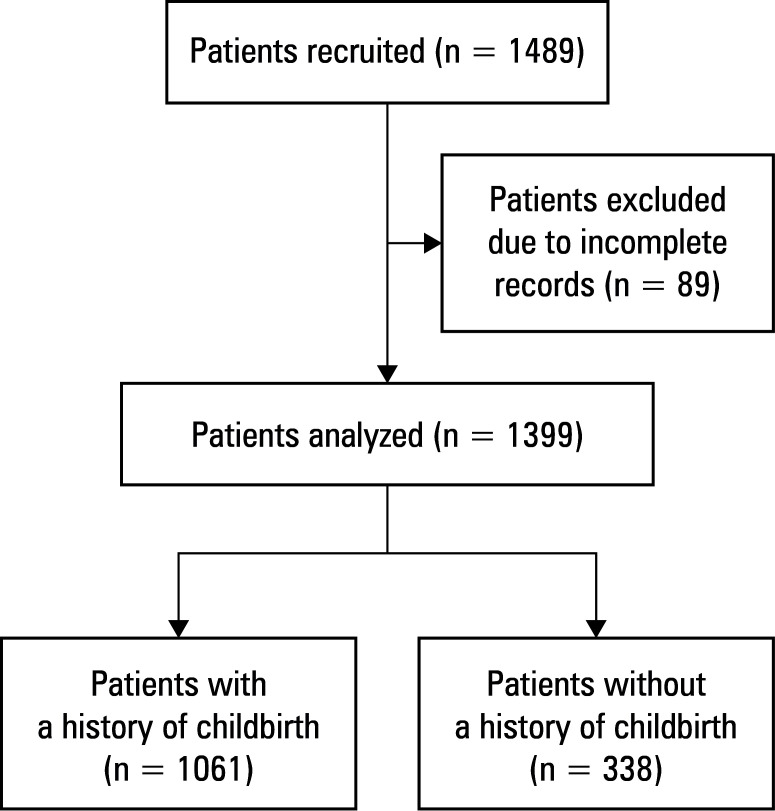
Patient selection flowchart

**TABLE 1:** Baseline characteristics of the study groups

Variable		History of pregnancy (n = 1061)	No history of pregnancy (n = 338)	P value
Age, y		42 (36–49)	32.5 (27–41)	<⁠0.001
Preoperative characteristics	Body weight, kg	118 (107–129)	124 (112–140)	<⁠0.001
	BMI, kg/m2	42.45 (39.52–46.28)	44.3 (40.64–49)	<⁠0.001
	T2DM	229 (21.6)	41 (12.1)	<⁠0.001
	Hypertension	476 (44.9)	79 (23.4)	<⁠0.001
	Hypercholesterolemia	416 (39.2)	43 (12.7)	<⁠0.001
	Metabolic syndrome	397 (37.4)	53 (15.7)	<⁠0.001
Type of surgery	SG	898 (76.4)	277 (82.)	0.23
	RYGB	152 (14.3)	56 (16.6)	
	OAGB	11 (1)	4 (1.2)	
	SASI	0	1 (0.3)	
Follow-up, y		1 (1–3)	1 (1–2)	0.11

Data are presented as number (percentage) or median (interquartile range).

Abbreviations: BMI, body mass index; OAGB, one anastomosis gastric bypass; RYGB, Roux-en-Y gastric bypass; SASI, single anastomosis sleeve ileal bypass; SG, sleeve gastrectomy; T2DM, type 2 diabetes mellitus

**TABLE 2:** Pregnancy / delivery history of the study population

Variable		Value
Number of pregnancies		2 (1–2)
Number of deliveries		1 (1–2)
History of miscarriages		115 (8.2)
Type of delivery	Natural	541 (51)
	Caesarean section	520 (49)
Number of caesarean sections performed		1 (0–1)
Causes for caesarean section	Fetal-related	237 (22)
	Maternal-related	324 (31)
	Labor-related	500 (47)
Breastfeeding	No	207 (20)
	<⁠6 months	454 (42)
	≥6 months	400 (38)
Time between last delivery and operation, mo		11 (5–20)

Data are presented as number (percentage) or median (interquartile range).

## INTRODUCTION

Obesity is a global health crisis, with rates having tripled in recent decades, according to the World Health Organization. ^1^ It increases the risk of serious conditions, such as type 2 diabetes mellitus (T2DM), cardiovascular disease, and cancer, reducing quality of life and raising mortality. While metabolic and bariatric surgery (MBS) is effective, weight loss outcomes vary, highlighting the need to understand factors influencing these differences to improve results. According to the 9th Global Registry Report of the International Federation for the Surgery of Obesity and Metabolic Disorders, the majority of patients undergoing metabolic procedures are women. ^2^ The annual number of bariatric surgeries in Poland is increasing rapidly. ^3^**,**^4^ Identifying predictors of weight loss success can help refine patient selection, improve preoperative counseling, and guide postoperative management strategies.

One area of growing interest is the influence of patients’ obstetric history, particularly childbirth, on weight loss outcomes following MBS. Pregnancy and childbirth lead to various physiological changes in a woman’s body, including significant weight gain, increased adiposity, and alterations in metabolic and hormonal profiles. During pregnancy, the body undergoes adaptive changes, such as increased insulin resistance, altered lipid metabolism, and shifts in estrogen and progesterone levels, to facilitate fetal development. ^5^**,**^6^ These changes can persist even after delivery, potentially affecting a woman’s ability to lose weight through traditional methods and surgical interventions. ^7^

Several studies have explored this topic, with mixed findings. ^8^**,**^9^**,**^10^**,**^11^ Some investigations suggest that women who have given birth may lose less weight after surgery than those who have never been pregnant, possibly due to residual hormonal imbalances or persistent metabolic changes that hinder weight loss. ^12^**,**^13^**,**^14^ Conversely, other studies have found no significant differences in weight loss outcomes between women with and without a history of childbirth, indicating that the role of obstetric history might be more complex and influenced by additional factors, such as age, number of pregnancies, interval between childbirth and surgery, and pre-existing metabolic conditions. ^15^**,**^16^

## AIM

This study aimed to elucidate the mechanisms through which childbirth may influence MBS outcomes and identify specific factors that mediate this relationship.

## MATERIALS AND METHODS

In this retrospective observational study, we analyzed women from 11 bariatric centers in Poland who underwent MBS in a tertiary referral bariatric center. It is a part of the multicenter project MOMBARIS (Maternal Outcomes of Bariatric Surgery and Pregnancy Study). ^17^ As an indication for surgical treatment, we used the recommendations of the Metabolic and Bariatric Surgery Chapter of the Association of Polish Surgeons, that is, body mass index (BMI) equal to or greater than 40 kg/m^2^ or 35 kg/m^2^ with obesity-related complications. ^18^ Inclusion criteria comprised: age of 18–65 years, eligibility for bariatric treatment, medical records including pregnancy and childbirth history, and 1-year follow-up. Patients were excluded if there was a change to a different procedure or necessary data were lacking. The study was designed and described taking into account all STROBE checklist points for observational studies. ^19^ Bariatric results are presented as percentage of weight loss, percentage of excess weight loss (%EWL), and percentage of total weight loss (%TWL). ^20^ The outcomes of MBS were described according to the standardized outcomes reporting. ^21^ The patients were divided into 2 groups: group 1 (with a history of pregnancy) and group 2 (without a history of pregnancy). The primary end point was to compare bariatric results between the groups. The data were collected retrospectively, hence no loss to follow-up information is available.

**TABLE 3:** Outcomes of the study groups

Variable	History of pregnancy (n=1061)	No history of pregnancy (n=338)	P value
%EWL	72.17 (53.45–87.8)	78.44 (58.07–95.14)	0.001
%TWL	28.57 (22.41–35.35)	33.85 (26.09–40.71)	<⁠0.001
%WL	33.2 (26–43)	41 (31–54)	<⁠0.001
Body weight after 1 year, kg	82 (74–94)	82 (73–92)	0.43
BMI after 1 year, kg/m2	29.76 (26.96–33.91)	29.41 (25.7–33.66)	0.08
Remission of T2DMa	167 (72.9% of the patients with preoperative T2DM)	35 (85.4% of the patients with preoperative T2DM)	0.3
Remission of hypertensiona	299 (62.82% of the patients with preoperative hypertension)	65 (82.25% of the patients with preoperative hypertension)	0.001
Remission of metabolic syndromea	239 (60.2% of the patients with preoperative metabolic syndrome)	27 (50.9% of the patients with preoperative metabolic syndrome)	0.77
Remission of hypercholesterolemiaa	220 (52.9% of the patients with preoperative hypercholesterolemia)	22 (51.2% of the patients with preoperative hypercholesterolemia)	0.94

Data are presented as number (percentage) or median (interquartile range).

a Percentage values are calculated in relation to the number of patients with a preoperative diagnosis of the disease.

Abbreviations: %EWL, percentage of excess weight loss; %TWL, percentage of total weight loss; %WL, percentage of weight loss; others, see TABLE 1

**TABLE 4:** Results of a multivariate regression model investigating influence of selected parameters on %EWL^a^

Variable	Value	SE	P value
Age	–0.33	0.07	<⁠0.001
Preoperative BMI	–1.19	0.11	<⁠0.001
T2DM	0.27	0.96	0.78
Hypertension	1.75	0.87	0.04
Dyslipidemia	2	0.86	0.02
Metabolic syndrome	0.91	1.02	0.37
Delivery before operation	1.4	0.86	0.11
SG	–11.87	6.57	0.07
RYGB	–7.96	6.67	0.23
OAGB	4	8.03	0.62

**a** Adjusted *R2* =13.45%; *P* <⁠0.001

Abbreviations: see TABLE 1

### Statistical analysis

Quantitative characteristics are presented as medians with interquartile ranges (IQRs). All quantitative characteristics were skewed data. The comparison of the patients’ baseline characteristics was conducted using the Mann–Whitney test for quantitative variables, and the χ^2^ test with or without the Yates correction was used for qualitative variables. A *P* value below 0.05 was considered significant. A multivariate regression all-effects model was built to test the influence of selected parameters on %EWL. The data were analyzed using Statistica software, version 12.0 PL (TIBCO Software Inc., Palo Alto, California, United States).

All procedures performed in this study adhered to the ethical standards of the institutional and national research committee and the 1964 Helsinki Declaration and its later amendments or comparable ethical standards. The study was approved by the Bioethics Committee of the Jagiellonian University (1072.6120.325.2022 and 118.00431.43.2025).

### RESULTS

Data from 11 bariatric centers involving 1488 patients were collected. After removing incomplete records, 1399 patients were included in the analysis. The study flowchart is presented in Figure 1. The patients without a history of pregnancy were younger (median [IQR] age, 32.5 [27–41] vs 42 [36–49] y; *P* <⁠0.001) and had lower incidence of T2DM (12% vs 22%; *P* <⁠0.001), hypertension (23.4% vs 44.9%; *P* <⁠0.001), and metabolic syndrome (15.7% vs 37.4%; *P* <⁠0.001) than those with a history of childbirth. The preoperative BMI was higher in the nulliparous women (median [IQR], 44.3 [40.64–49] vs 42.45 [39.52–46.28] kg/m^2^ ; *P* <⁠0.001; TABLE 1).

The data on the history of pregnancies and deliveries are outlined in TABLE 2.

Weight loss was less pronounced in group 1 ( TABLE 3 ). The %TWL in this group was by 15% lower (median [IQR], 28.57% [22.41%–35.35%] vs 33.85% [26.09%–40.71%]), whereas the %EWL was worse by 8% (median [IQR], 72.17% [53.45%–87.8%] vs 78.44% [58.07%–95.14%], as compared with group 2. Both groups achieved similar BMI at 1-year follow-up (median [IQR], 29.76 [26.96–33.91] vs 29.41 [25.7–33.66] kg/m^2^ in groups 1 and 2, respectively). The rate of remission of obesity-related diseases was similar in both groups.

A multivariate regression all-effects model was built to test the influence of selected parameters on %EWL. The results are contained in TABLE 4 . The factors that altered %EWL were: age ( *P* <⁠0.001), preoperative BMI ( *P* <⁠0.001), arterial hypertension ( *P* =0.04), and preoperative dyslipidemia ( *P* =0.02). A history of childbirth was not an independent factor in the model.

### Discussion

Weight loss following MBS is well documented, yet the variability in outcomes highlights the influence of diverse factors, including demographic, metabolic, and lifestyle variables. This study adds to the growing body of literature by focusing on the impact of pregnancy history, demonstrating that women with prior childbirth achieve significantly lower weight loss metrics, such as %EWL and %TWL, as compared with their nulliparous counterparts.

Our findings align with those of Froylich et al, ^11^ who reported a marked reduction in %EWL in 62 postpartum patients, as compared with 92 nulliparous women. %EWL following pregnancy in the group with a history of childbirth was 53%, whereas in the women without such history, it was 68%. The difference in weight loss outcomes in our study is less pronounced, likely due to a larger sample size. Conversely, Hecht et al ^22^ found no significant association between pregnancy history and weight loss, emphasizing the complexity of this relationship. Their study involved 250 women, in whom weight loss outcomes were generally worse in comparison with the data presented herein.

These discrepancies may stem from variations in sample sizes and baseline patient characteristics. The differences in weight loss outcomes may be attributed to many different factors that yield significant results when combined. One key factor is age, which differed markedly between the groups in our study. It has been consistently associated with weight loss outcomes, ^23^**,**^24^**,**^25^**,**^26^**,**^27^**,**^28^**,**^29^ as demonstrated by Pfefferkorn et al ^24^ and Ochner et al, ^25^ who observed a decline in %EWL with increasing age. Additionally, metabolic changes induced by pregnancy, including insulin resistance and altered fat metabolism, may persist after delivery and impede weight loss. Davis et al ^30^ highlighted the lasting impact of gestational weight gain on long-term obesity risk, while Berggren et al ^31^ suggested that the reversibility of pregnancy-related metabolic changes depends on postpartum weight recovery.

The large sample size in this multicenter study strengthens the reliability of our findings. However, the retrospective design and incomplete data on the time between pregnancy and surgery limit our ability to draw causal inferences or assess the temporal influence of pregnancy on MBS outcomes. Future studies should adopt a prospective approach, incorporating detailed metabolic assessments and controlling for age to better elucidate these relationships.

### CONCLUSIONS

Patients with a history of childbirth exhibit worse weight loss outcomes after MBS than nulliparous women; however, childbirth is not an independent factor hindering weight loss. Notably, %EWL remains clinically significant in this group, exceeding 70%. These findings underscore the need for individualized patient counseling and further research into optimizing outcomes for postpartum patients undergoing MBS.
